# Repair of DNA Double-Strand Breaks in Heterochromatin

**DOI:** 10.3390/biom6040047

**Published:** 2016-12-16

**Authors:** Felicity Z. Watts

**Affiliations:** Genome Damage and Stability Centre, School of Life Sciences, University of Sussex, Falmer, Brighton BN1 9RR , UK; f.z.watts@sussex.ac.uk; Tel.: +44-1273-678257

**Keywords:** HP1, 53BP1, BRCA1, γH2AX, homologous recombination, c-NHEJ, alt-NHEJ

## Abstract

DNA double-strand breaks (DSBs) are among the most damaging lesions in DNA, since, if not identified and repaired, they can lead to insertions, deletions or chromosomal rearrangements. DSBs can be in the form of simple or complex breaks, and may be repaired by one of a number of processes, the nature of which depends on the complexity of the break or the position of the break within the chromatin. In eukaryotic cells, nuclear DNA is maintained as either euchromatin (EC) which is loosely packed, or in a denser form, much of which is heterochromatin (HC). Due to the less accessible nature of the DNA in HC as compared to that in EC, repair of damage in HC is not as straightforward as repair in EC. Here we review the literature on how cells deal with DSBs in HC.

## 1. Introduction

DNA double-strand breaks (DSBs) pose serious threats to genetic integrity and cell viability, since, if not identified and repaired, they can lead to insertions or deletions (indels) or gross chromosomal rearrangements. They can be simple blunt-ended breaks or more complex breaks such as DSBs with incompatible termini, clustered oxidative lesions (base damage, crosslinks, etc.) or breaks that are in close proximity to other single-strand breaks (SSBs) or DSB lesions. They are produced by a variety of different agents. For example, ionising radiation (IR) and high-energy linear energy transfer (LET) radiation can produce complex breaks, while some DSB repair assays, dependent on the activity of site-specific nucleases such as I-SceI, create simple breaks. There are several processes in cells for the repair of DSBs, the choice of which is dependent partly on the nature of the break, and partly on the location of the break within the chromatin.

**Figure 1:**
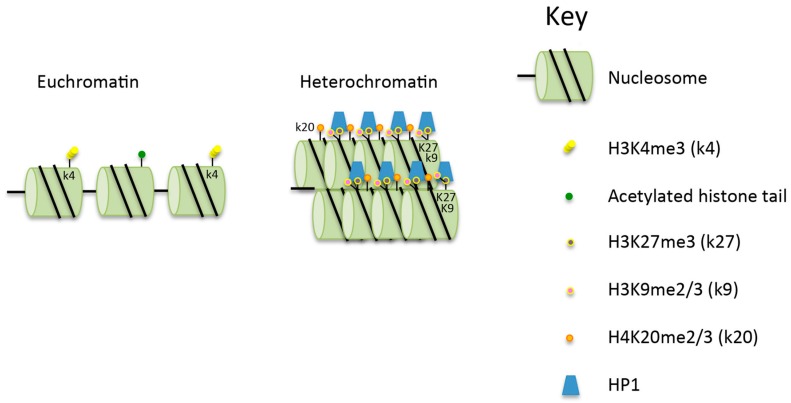
Comparison of the structure of euchromatin and heterochromatin. Heterochromatin is hypoacetylated and hypermethylated on histone H3K9 (H3k9me2/3 (k9), H3k27me3 (k27) and H4k20me2/3 (k20) compared to euchromatin. See text for further details. HP1: heterochromatin protein 1.

Eukaryotic DNA is composed of euchromatin and heterochromatin, with the majority of the chromatin being maintained as euchromatin. The relative amounts of euchromatin (EC) and heterochromatin (HC) in cells depend on a range of factors, including cell types, cell age and gender [1]. Euchromatin contains the majority of the transcribed genes, and is a loosely packed form that allows access to the DNA by proteins including transcription factors which control gene expression. In contrast, heterochromatin is a more tightly packed form of chromatin (Figure 1) [2,3] where there is limited access to transcription factors and DNA repair proteins. The aim here is to review the literature on the repair of DSBs in heterochromatin, focusing on repair in mammalian cells, unless otherwise stated.

## 2. The Structure, Importance and Maintenance of Heterochromatin

Heterochromatin has several specific and important roles in cells. Contained within it are the centromeres, pericentromeric regions, telomeres and highly repetitive sequences. These regions comprise constitutive HC, while silenced and/or developmentally regulated genes make up the facultative HC [4]. Constitutive HC contains histones that are generally hypoacetylated and hypermethylated at histone H3 lysine 9 (H3K9me2/3) and lysine 27 (H3K27me3), as well as at histone H4 lysine 20 (H4K20me2/3) [5] (Figure 1). These marks have been demonstrated to be associated with tethering of the chromatin to the nuclear lamina [6]. The processes associated with the creation of the histone modifications, and the maintenance of HC requires a large number of proteins, including the histone modifiers SET domain bifurcated 1 (SETDB1) and methyltransferase suppressor of variegation 3–9 (SUV39), both of which are required for methylation of H3K9. Also required are histone deacetylases (HDACs) [7] and heterochromatin protein 1 (HP1) which recognises and interacts with H3K9me2/3 (Figure 1). Two other proteins which affect chromatin structure, and which are relevant to the discussion of DSB repair in HC, are KRAB domain associated protein 1 (KAP-1) which interacts with HP1, and SWI/SNF-related matrix-associated actin-dependent regulator of chromatin, subfamily A, containing DEAD/H box 1 (SMARCAD1). KAP-1 has multiple cellular functions brought about through its ability to maintain chromatin compaction. It is recruited to HC via KRAB domain-containing, DNA sequence-specific repressor proteins [8,9]. Additionally, it interacts with HP1 and subsequently with SETDB1 [5,10], histone deacetylase 1 (HDAC1) and histone deacetylase 2 (HDAC2), and the nucleosome-remodeling factor CHD3 isoform 1 (CHD3.1) [11]. SMARCAD1 acts at replication sites to facilitate the deacetylation of newly assembled histones and thus acts to maintain correct silencing [12]. It has also been proposed to weaken histone–DNA interactions in nucleosomes flanking DSBs, facilitating the resection of DNA at DSBs in preparation for homologous recombination (HR) [13]. SMARCAD1 knock-down reduces the level of methylation of H3K9 and results in the delocalisation of HP1, HDAC1 and KAP-1 from the chromatin [14].

## 3. Repair of DSBs

**Figure 2:**
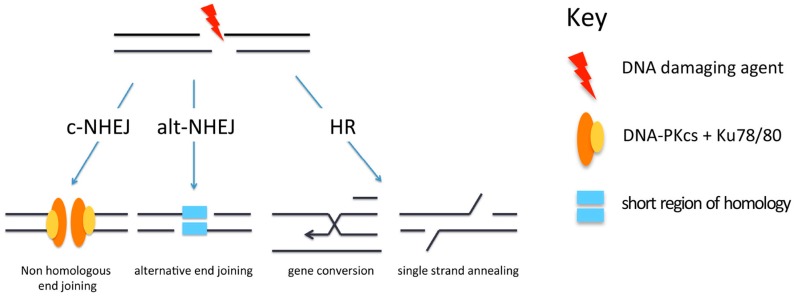
Pathways for repair of DNA double-strand breaks (DSBs). Repair of DSBs can occur by canonical non-homologous end-joining (c-NHEJ), alternative non-homologous end-joining (alt-NHEJ) or homologous recombination (HR). DNA-PKcs: DNA-dependent protein kinase catalytic subunit

Several pathways are available for the repair of DSBs, and these include canonical non-homologous end-joining (c-NHEJ), alternative non-homologous end-joining (alt-NHEJ) and homologous recombination (HR) (Figure 2). The repair pathway that is used is influenced by the nature of the break. Since these repair processes have been reviewed extensively recently [15,16,17], they will simply be summarised here. c-NHEJ is the main repair pathway used for the repair of simple DSBs in G1, since G1 cells have no sister chromatids so that HR is not an option. In S or G2 cells, when a sister chromatid is present, it might intuitively be expected that HR would be used as this is a relatively error-free process when compared to c-NHEJ. However, a number of studies have shown that the first choice of repair pathway in S or G2 is in fact c-NHEJ [18].

**Figure 3:**
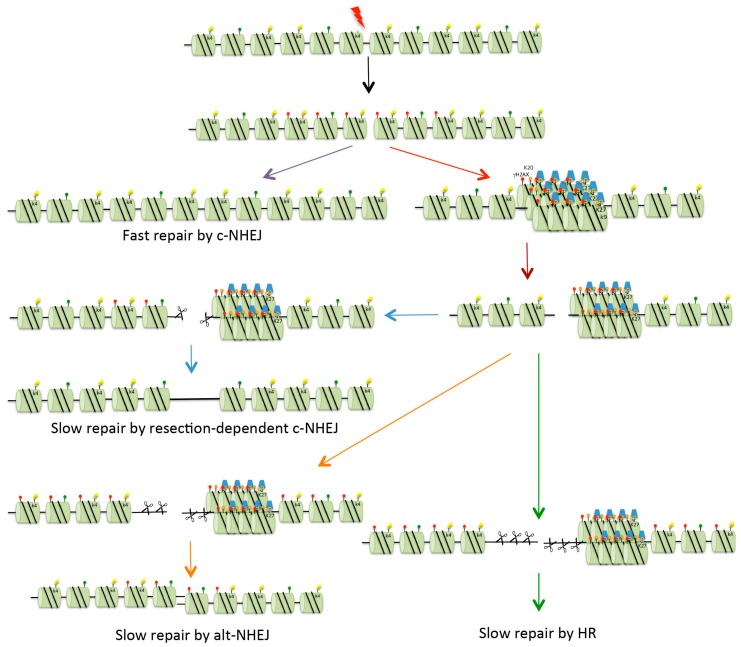
Repair of DSBs in heterochromatin. Four possible mechanisms exist for the repair of DSBs. Fast c-NHEJ (purple arrow) involves the religation of broken ends in a Ku70/80-dependent process. DSBs that cannot be repaired quickly might be those present in heterochromatin (HC), or might be breaks occurring in euchromatin that are sequestered into HC for protection (red arrow). In order for repair to take place, the DSBs are relocated on the surface of the HC (brown arrow). These breaks can be repaired with slow kinetics by resection-mediated c-NHEJ, involving the nuclease Artemis (blue arrows). In mice and under certain conditions (see text for details), DSBs can be repaired by alt-NHEJ (orange arrows). In G2, if NHEJ is unable to repair DSBs, there is further resection with repair occurring via HR (either gene conversion or single-strand annealing, see Figure 2) (green arrows). Red dots indicate histone H2AX phosphorylation (γH2AX). For key to other histone modifications see Figure 2.

The first events following the creation of a DSB are the recognition of the break by the Ku70/80 heterodimer and the Mre11/Rad50/Nbs1 (MRN) complex. The Ku70/80 heterodimer interacts with DNA and functions to hold together the two DNA ends. This occurs very rapidly after the creation of the DSB, and the resulting complex acts as a scaffold to recruit further c-NHEJ factors. The first protein to be recruited is DNA-dependent protein kinase catalytic subunit (DNA-PKcs), a protein that is capable of phosphorylating numerous repair factors, and this is followed by DNA ligase 4 (LIG4), X-ray repair cross complementing protein 4 (XRCC4) and XRCC4-like factor (XLF). This series of events is responsible for fast kinetics repair in G1 and is used for the repair of simple DSBs. If these proteins are unable to repair the DSB, perhaps due to the presence of complex breaks or the location of the break in the chromatin, a battery of other proteins is recruited. One of these is the nuclease Artemis [19] which brings about limited resection of the DNA ends to allow end ligation (Figure 3). This repair process is termed resection-dependent c-NHEJ [20]. In mice, and to a lesser extent in humans (e.g., in cells that are deficient in Ku, knocked down for p53 binding protein 1 (53BP1) or in some tumour cells), damage can be repaired by alt-NHEJ [21]. This process involves C-terminal binding protein (CtBP), interacting protein (CtIP) [22] and DNA ligase 3α (LIG3α) [23].

**Figure 4:**
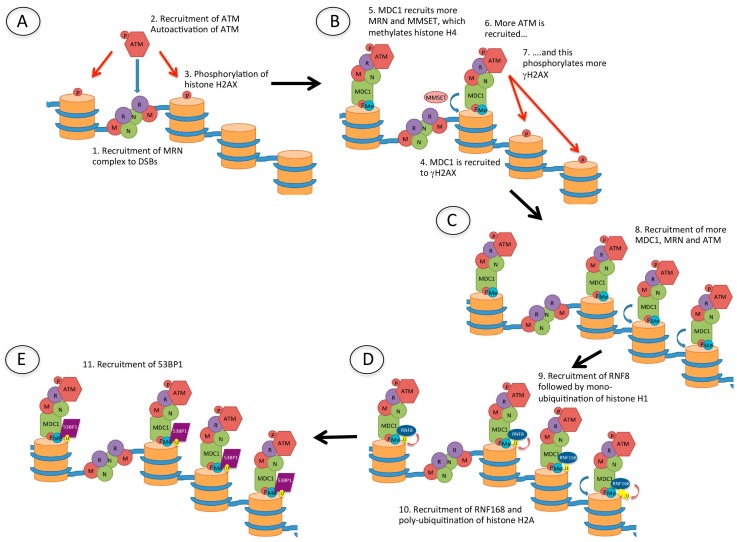
Recruitment of p53 binding protein 1 (53BP1) to DSBs. (**A**) Following the creation of a DSB, the Mre11/Rad50/Nbs1 (MRN) complex is recruited to the DNA ends. MRN recruits ataxia-telangiectasia mutated (ATM), which is autophosphorylated and phosphorylates (red arrow) histone H2AX (γH2AX); (**B**) Mediator of DNA damage checkpoint 1 (MDC1) is then recruited to γH2AX, and this recruits more MRN, as well as multiple myeloma SET domain (MMSET) protein which methylates histone H4. MDC1 recruits more ATM and this amplifies the γH2AX signal; (**C**) The damage signal is further amplified by recruitment of more MDC1, MRN and ATM which phosphorylates further histone H2AX; (**D**) The ubiquitin E3 ligases RNF8 and RNF168 are then recruited, resulting in mono-ubiquitination of histone H1 and poly-ubiquitination of histone H2, respectively; (**E**) 53BP1 is finally recruited to methylated histone H4 (H4K20me2) and ubiquitinated histone H2A (H2AK15Ub).

MRN, another complex that is rapidly recruited to DSBs, recruits ataxia-telangiectasia mutated (ATM) which phosphorylates histone H2AX (γH2AX) (Figure 4). This phosphorylation event occurs within seconds of the creation of the DSB, and spreads over hundreds of thousands to millions of bases surrounding the site of damage [24] and acts as a signal for DNA damage. In turn, γH2AX recruits mediator of DNA damage checkpoint 1 (MDC1), which then recruits more MRN and ATM, further propagating the γH2AX DNA damage signal. Also recruited are a number of ubiquitin E3 ligases: RNF8 and RNF168, leading to ubiquitination of histones H1 (likely on several lysine residues [28]) and H2A (H2AK13/15Ub) [25,26,27], as well as RNF20 and RNF40 which ubiquitinate histone H2B [28]. RNF20 and RNF40 both interact with, and are phosphorylated by ATM [29]. The recruitment of these proteins to DSBs produces what have been termed ionising radiation-induced foci (IRIF). Following these events, 53BP1 is recruited through interaction of its tudor domain with H4K20me2 (a constitutive histone mark), and of its ubiquitination-dependent recruitment (UDR) domain with H2AK15Ub [30]. The importance of this will be discussed below.

In G2 cells, if c-NHEJ fails to repair DSBs, HR is invoked (Figure 3). HR involves more extensive end-processing than that occurring during alt-NHEJ in that it requires long overhanging 3’ DNA ends for strand invasion of an undamaged template to promote repair [16] (Figure 2). Results from several labs have implicated 53BP1 as having a role in controlling the pathway choice, proposing that it promotes NHEJ and inhibits HR, at least at early times after the creation of a DSB [31,32]. It does this by recruiting factors such as replication timing regulatory factor (RIF1), REV7, PAX-interacting protein (PTIP) and Artemis [31,33,34,35] to prevent extensive end resection. In addition to this inhibitory role, it has been recently demonstrated that 53BP1 has a role in promoting the error-free (gene conversion) form of HR [36]. Here it allows some limited resection to produce 3’ overhanging ends that are recognised by Rad51 recombinase, resulting in repair by gene conversion. In the absence of 53BP1, there is extensive resection and this leads to the recruitment of Rad52, thus promoting single-strand annealing. Following resection, breast cancer susceptibility gene 1 (BRCA1) is recruited to IRIF, and 53BP1 is relocalised to the periphery of the foci [37,38]. This relocalisation requires the BRCA1 C-terminal (BRCT) domains of 53BP1.

## 4. DSBs and Heterochromatin

A number of studies have demonstrated that chromatin organisation has a striking effect on mutation rates, with rates being significantly higher in HC compared to levels in EC [39,40]. The reason for this is not clear, and could be accounted for by a number of factors associated with HC, such as different accessibility to DNA repair factors, difficulty in signaling the presence of DNA damage or increased sensitivity to mutagenic agents.

### 4.1. Do DSBs Occur with Equal Frequency in EC and HC?

The idea that DNA within HC is more susceptible to damage goes against current theories, since, as well as maintaining particular chromosomal structures and transcriptional silencing, one of the roles of HC has been proposed to be the protection of DNA against damage or inappropriate recombination events [41]. Protection against unscheduled HR is particularly important in the case of DSBs occurring in the highly repeated sequences in the ribosomal DNA (rDNA) and pericentromeric regions, since such repair could lead to insertions, deletions, and chromosomal rearrangements. The notion of a protective role for HC is supported by a recent study analysing the production and repair of DSBs in human embryonic stem cells (hEScs) where the chromatin has a more open structure than that in differentiated cells [42]. It was observed that the same dose of radiation produced significantly more 53BP1-containing IRIF in hESCs than in normal fibroblasts, from which the authors concluded that DNA within HC may indeed be protected against damage. These results need to be tempered by the fact that the formation of IRIF might be suppressed in HC, or that DSBs might be repaired with faster kinetics in cells with less heterochromatin, making the presence of IRIF an imperfect measure of the amount of damage. Additionally, the redox status can vary in different cell lineages, and thus cells with reduced ability to absorb reactive oxygen species may be more susceptible to DNA damage (e.g., [43,44]).

### 4.2. Once Created Are DSBs Protected in Heterochromatin?

As well as having a protective role in preventing DNA damage from occurring in the first place, it has been proposed that HC protects DNA against further damage once a DSB has been created. While this area needs further research, it has been demonstrated that HP1 accumulates at sites of DNA damage [45,46]. The outcome of this might be that damaged DNA is corralled into HC to reduce access to potentially harmful nucleases, in order to limit the extent of the damage. More recently it has been proposed that HP1 binding to damaged DNA helps stabilise ends and keep sister chromatids together [47].

### 4.3. Repair of DSBs in Heterochromatin

It has been known for some time that higher-order chromatin packaging acts as a barrier to detection and repair of DNA damage [48]. A number of studies have demonstrated that chromatin undergoes conformation changes following the creation of damage. One such study indicates that following the creation of laser-induced DNA damage, there is rapid expansion of chromatin around the site of irradiation [49]. Although this occurs with the same kinetics in EC and HC, subsequent treatment of damage sites is different. For example, after exposure to IR the majority of γH2AX foci are located outside of, or close to, HC [50,51]. It has also been demonstrated that in *Drosophila*, some breaks migrate to the nuclear periphery for repair by HR [52] (Figure 3). HC is very dynamic, and so this movement is likely due to Brownian motion as no genetic component has been identified as being involved. This localisation at the nuclear periphery would be consistent with H3K9me being associated with tethering to the nuclear lamina [6]. Recently it has been proposed that following the creation of a DSB in EC, there is a transition to a more closed form of chromatin, via the transient formation of an HP1-dependent HC-like chromatin domain [53].

### 4.4. Role of 53BP1 in Repair of DSBs in Heterochromatin

DSBs can be repaired with either fast or slow kinetics (Figure 3). Slow kinetics repair requires 53BP1, and this has been proposed to be the repair of DSBs in HC [54]. In a study using mutants that accumulate unprotected breaks at telomeres (structures maintained within HC), it was demonstrated that 53BP1 has a role in increasing chromatin mobility to promote c-NHEJ [55]. Further, Noon et al. demonstrated that IR-generated DSBs in HC are repaired with slower kinetics than DSBs in EC [56]. Specifically, they demonstrated that in G1, 53BP1 is required at late times to concentrate ATM at unrepaired DSBs in order to phosphorylate KAP-1. In undamaged heterochromatin, sumoylated KAP-1 interacts with a SUMO-interacting motif (SIM) in CHD3.1, which is a component of a nucleosome remodeling complex [57]. Phosphorylation of KAP-1 disrupts this SUMO:SIM interaction, causing CHD3.1 to be released from the chromatin [58]. Following the release of CHD3.1, the compacted chromatin needs to be relaxed. This requires the ATP-dependent chromatin assembly factor large subunit 1 (ACF1)–sucrose non-fermentable protein 2 homolog (SNF2H) chromatin remodeling complex, which is dependent on the E3 ubiquitin ligases RNF20 and RNF40 for activity [59]. While the KAP-1 and CHD3.1 functions are independent of ACF1–SNF2, both are dependent on ATM phosphorylation [58,59]. Recent studies have demonstrated that the maintenance of ATM at slow-repairing DSBs in G1 requires the phosphopeptide-binding site (PPBS) of the 53BP1 BRCT domains [60]. It was demonstrated that the PPBS binds γH2AX; however, how this affects ATM tethering remains to be determined. As well as a requirement for the relaxation of HC via the release of KAP-1 from HC, there is also evidence that some aspect of chromatin compaction is required for efficient HR, since knock-down of HP1, HDAC1/2, SUV39 or SETDB1 is required for BRCA1 function in repositioning 53BP1 during HR [61,62].

### 4.5. Role of BRCA1

BRCA1 is another protein recruited to DSBs throughout the genome: it has a pivotal role in HR if NHEJ is unable to repair the damage [37,38]. In order to undertake its myriad of functions, it acts as a molecular scaffold and forms a number of complexes. One of these is with Abraxas and receptor-associated protein 80 (RAP80) [63]. This complex is retained at the damage site via the RNF8/RNF168-mediated polyubiquitin chains and acts to repress resection and promote NHEJ [64]. Another one of BRCA1’s interacting partners is BRCA1-associated RING domain protein 1 (BARD1): together they constitute an ubiquitin ligase [65]. Like the BRCA1/Abraxas/RAP80 complex, the BRCA1–BARD1 complex is also recruited at early times to damage sites; in this case it is via the BRCT domain of BARD1 which interacts with poly-ADP ribose (PAR) [66]. Since PAR is a transient signal, the BRCT domain of BARD1 becomes available for further interactions at later times. After further resection and commitment to HR, BRCA1 is found in IRIF, with 53BP1 displaced to a position peripheral to the BRCA1 foci [56]. Recently, this late recruitment of BRCA1 to sites of damage has been demonstrated to depend on HP1, suggesting a more specific role in the repair of damage in HC. More specifically, its recruitment depends on an ATM-dependent interaction of BARD1 and H3K9me2 [67,68,69]. This involves the interaction of a conserved motif in the BRCT domain of BARD1 with the chromoshadow domain of HP1 [69]. Following commitment to HR, the ubiquitin ligase activity of the heterodimer is used to modify histone H2A lysine 127 (H2AK127Ub) [70]. This in turn recruits the nucleosome remodeler SMARCAD1. Interestingly, the kinetics of recruitment of SMARCAD1 to DSBs are the same as that of exonuclease 1, consistent with a role for SMARCAD1 in HR [71].

## 5. Summary

Due to the compacted nature of HC compared to EC, it is clear that different mechanisms are needed to signal and repair DSBs in these two types of chromatin. As HC comprises many different types of chromosomal regions, it would not be unexpected that the repair of damage in different regions involves different processes. For example, it has been observed that DSBs in pericentromeric DNA are treated differently compared to those within centromeres [72], and that repair in the rDNA and fragile sites is likely to be different than that in centromeres and centromeric regions [73]. Further work will be needed to fully elucidate the repair mechanisms utilised and how they are regulated. In summary, 53BP1 and BRCA1, key proteins influencing how DSBs are repaired, both have functions that are dependent on HC and HC-interacting proteins. The data provide evidence for a complex role for HC in the recognition, response to and repair of DSBs, and that extensive chromatin remodeling is likely to be required for the repair of many of the DSBs that occur in cells.
